# The Effect of Ethnicity on 2D and 3D Frontomaxillary Facial Angle Measurement in the First Trimester

**DOI:** 10.1155/2013/847293

**Published:** 2013-10-28

**Authors:** Jennifer Alphonse, Jennifer Cox, Jill Clarke, Philip Schluter, Andrew McLennan

**Affiliations:** ^1^The University of Sydney, Faculty of Health Sciences, P.O. Box 170, Lidcombe, NSW 1825, Australia; ^2^Sydney Ultrasound for Women, Suite 114, Level 1, Q Central, 10 Norbrik Drive, Bella Vista, NSW 2153, Australia; ^3^The University of Canterbury, School of Health Sciences, Private Bag 4800, Christchurch 8140, New Zealand; ^4^The University of Queensland, School of Nursing and Midwifery, Level 2, Edith Cavell Building, UQ, Herston Campus, Qld 4029, Australia; ^5^The University of Sydney, Faculty of Medicine, Camperdown, NSW 2066, Australia

## Abstract

*Objectives*. To determine the existence and extent of ethnic differences in 2D or 3D fetal frontomaxillary facial angle (FMFA) measurements. *Methods*. During routine 11–14 weeks nuchal translucency screening undertaken in a private ultrasound practice in Sydney, Australia, 2D images and 3D volumes of the fetal profile were collected from consenting patients. FMFA was measured on a frozen 2D ultrasound image in the appropriate plane and, after a delay of at least 48 hours, was also measured on the reconstructed 3D ultrasound volume offline. *Results*. Overall 416 patients were included in the study; 220 Caucasian, 108 north Asian, 36 east Asian and 52 south Asian patients. Caucasians had significantly lower median FMFA measurements than Asians in both 2D (2.2°; *P* < 0.001) and 3D (3.4°; *P* < 0.001) images. Median 2D measurements were significantly higher than 3D measurements in the Caucasian and south Asian groups (*P* < 0.001 and *P* = 0.04), but not in north and east Asian groups (*P* = 0.08 and *P* = 0.41). *Conclusions*. Significant ethnic variations in both 2D and 3D FMFA measurements exist. These differences may indicate the need to establish ethnic-specific reference ranges for both 2D and 3D imaging.

## 1. Introduction

Ultrasound-based screening for fetal aneuploidy in the first trimester has developed rapidly over the past 20 years. The original observation that faces of Down syndrome individuals were flat led to the investigation of frontomaxillary facial angle (FMFA) measurement as a risk factor for trisomy 21 [1]. Reference ranges for FMFA using offline three-dimensional (3D) reconstruction software were developed in the first trimester identifying the 95th percentile measurement as 85° [2]. In a previous study, we demonstrated that 2D FMFA measurements were similar, but not equivalent, to those obtained by the 3D method; thus, normative data would need to be collected from the normal population separately for both 2D and 3D [3]. Given the potential ethnic variations, normative data from local populations may also be necessary.

A recent study has cast doubt on the efficacy of first trimester FMFA measurement as a screening method for Down syndrome in an Asian population. Kwon and colleagues demonstrated that 3D FMFA measurements in the normal Korean population were substantially wider than the previously reported mean which would increase the screening false positive rate [4]. A study by Chen et al. [5] contradicts this finding in the Asian population, as the difference in FMFA measurements between Caucasian and Chinese patients was thought to be so small that it was not clinically significant.

Given the uncertainties relating to ethnic variation in FMFA measurement, the current study aims to compare the 2D and 3D FMFA measurements between Caucasian, North Asian, South Asian, and East Asian populations. 

## 2. Material and Methods

### 2.1. Design

This prospective study was conducted in a specialist private prenatal screening and diagnostic practice over two years. Observers certified in nuchal translucency (NT) measurement collected 2D images and 3D volumes of the fetal face.

### 2.2. Participants

Only consenting patients with singleton pregnancies referred for NT screening with a gestation between 11 + 0 and 13 + 6 weeks (defined by a crown-rump length (CRL) range of 45–84 mm) were included in this study. The patient's self-determined ethnicity was entered into the database. Patients from China, Hong Kong, Japan, Nepal, Taiwan and Korea were classified as North Asian. Patients from India, Pakistan, Sri Lanka, and Fijian Indians were classified as South Asian. Patients from Thailand, Vietnam, Singapore, Indonesia, Philippines, Laos, Cambodia, and Malaysia were classified as East Asian [6]. Patients from mixed ethnicity backgrounds, Pacific islands or where ethnicity was not determined were excluded. Other reasons for exclusion were (a) inadequate visualization of the fetal face due to maternal factors (increased maternal body habitus or uterine retroversion) or fetal position (erect, prone, or hyperflexed), (b) if the allocated time for the study (30 minutes) was exceeded without obtaining the correct fetal profile, and (c) fetuses with abnormal karyotype.

### 2.3. Instruments and Procedure

The examinations were performed on a Voluson E8 Expert (GE Medical Systems, Milwaukee, WI, USA) with a RAB4-8L probe. Standard measurements of the crown rump length and NT were performed in accordance with Fetal Medicine Foundation (FMF) guidelines (http://www.fetalmedicine.com/fmf/). 

**Figure 1:**
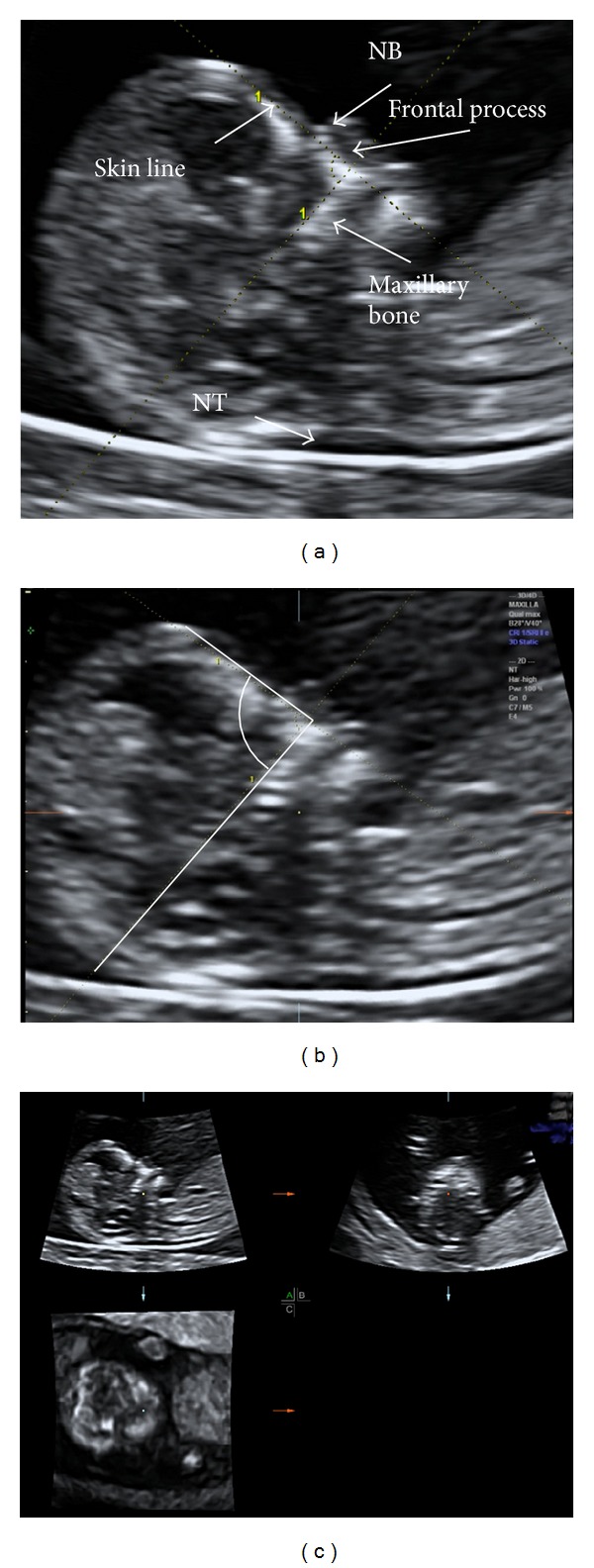
(a) 2D fetal profile with sonographic landmarks. (b) 3D reconstruction of the same fetus with caliper placement for FMF angle measurement. (c) 3D sectional planes of the same fetus.

The methods of image and volume collection have been previously described [2, 7]. In brief, the 2D image was collected when the fetus was in supine position and mid-sagittal plane. Visualization of the NT, nasal bone, skin line, frontal process of the maxillary bone, and maxillary bone is required (Figure 1(a)). The transducer must be at 90° to the fetal frontal bone to maximize ultrasound reflectivity. The first line is positioned in a plane incorporating the most anterior protrusion of the frontal bone (excluding skin) and the anterior tip of the maxillary bone, as determined by the frontal process of the maxillary bone. The second line is then placed superiorly along the most continuous and echogenic margin of the maxillary bone to intersect with the first line. The angle formed between these two lines is then recorded (Figure 1(b)).

The 2D FMFA angle was measured on the ultrasound machine during the course of the 11–14-week examination. The 2D image of the fetal profile and 3D volume of the fetal profile were both collected (Figure 1(c)). The 3D volume was sent via network connection to 4D view (GE Healthcare) for offline analysis, while the 2D image was saved onto a compact disk (CD) for later measurement by the same observer. The observer was blinded to the results, and both angle measurements were recorded in a database (Excel, Microsoft Corporation, http://www.microsoft.com/). 

### 2.4. Statistical Analysis

Patterns in the FMFA measurement data over time and gestation were investigated using scatter plots and histograms. Wilcoxon matched-pairs signed-ranks test was performed to assess the relationship between ethnicities and also to test for relationships between matched observations from 2D and 3D images. All statistical analyses were performed using Stata version 12.0 (Stata Corp, College Station, TX, USA), and a level of *α* = 5% was used to determine statistical significance. 

### 2.5. Ethical Clearance

Ethics approval was obtained from The University of Sydney Human Research Ethics Committee (HREC) approval number 13367. All information, images, and data were stored in accordance with HREC instructions.

## 3. Results

### 3.1. Sample Characteristics

Overall, 687 eligible patients were approached to participate in the study, of whom 620 (90.8%) consented. Six (6) patients were excluded with confirmed aneuploidy (trisomy 21, trisomy 13, trisomy 18, and Turners syndrome). The remaining fetuses were euploid as determined by prenatal testing or by postnatal follow-up consultation. 3D volume was not collected in 77 subjects due to maternal factors or because correct fetal position could not be obtained within the allotted scanning time. One hundred and twenty one did not have adequate ethnicity information in the NT risk factor analysis program. In the final cohort of 416, 220 (53%) patients were Caucasian, 108 (26%) were classified as North Asian, 52 (12%) as South Asian, and 36 (9%) as East Asian. All 220 Caucasian patients had both 2D and 3D FMFA measurements performed. As several operators were not specifically trained to perform FMFA measurement, only 95 (88%) North Asian, 47 (90%) South Asian, and 29 (81%) East Asian patients had 2D FMFA measurements performed. All patients had a 3D FMFA measurement recorded. The median patient age across all ethnicities was 31.5 years (range: 21 years to 44 years), and the median gestational age was 12 weeks and 4 days (median CRL 62.6 mm (range: 47 mm to 82 mm)) with no significant differences between the ethnic groups.

### 3.2. Frontomaxillary Facial Angle (FMFA) Measurements

**Figure 2:**
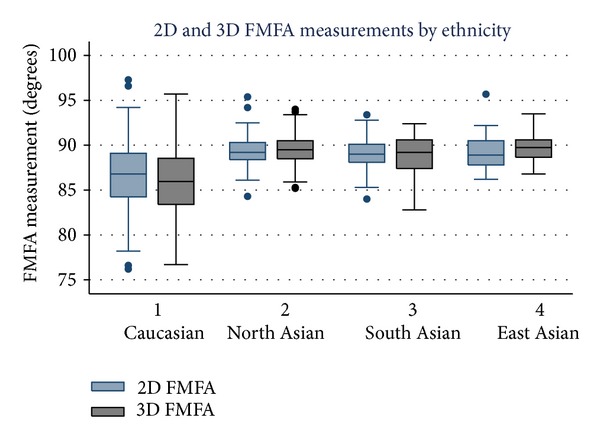
Box and whisker plot demonstrating the median, range, and variability of frontomaxillary facial angle measurement.

The Caucasian group had significantly smaller FMFA measurements in both 2D and 3D imaging when compared with all the Asian sub-groups (*P* < 0.001). No difference was found in 2D measurements between any of the Asian sub-groups. However, significant differences were found in 3D FMFA measurement between all Asian sub-groups, with North Asian fetuses having larger measurements than those of South Asian extraction (*P* = 0.04) and East Asian fetuses having larger measurements than both North Asian (*P* = 0.05) and South Asian (*P* = 0.01) fetuses. A difference was identified between 2D and 3D FMFA measurements in the Caucasian and South Asian groups (*P* = 0.0001 and *P* = 0.04, resp.) but not between the north and East Asian groups (*P* = 0.08 and *P* = 0.411, resp.). The median, range, and variability of FMFA measurements are presented in Figure 2, and all group comparisons are presented in Table 1.

In the Caucasian group, 31% had a 2D FMFA measurement greater than 85.0°, but, in contrast, 47% of North Asian, 77% of South Asian, and 76% of East Asian fetuses had 2D FMFA measurements greater than 85.0°. In 3D, the findings were similar, with 26% Caucasian, 49% North Asian, 58% South Asian, and 78% East Asian having a 3D FMFA measurement greater than 85°. Overall, 61% (2D) and 57% (3D) of the Asian group expressed FMFA measurements exceeding 85°. 

### 3.3. Time Taken to Perform Frontomaxillary Facial Angle (FMFA) Measurements

Collecting the 2D image and the 3D volume did not extend the duration of the examination. Caliper placement to measure the FMFA on the 2D image took approximately fifteen seconds. The average time to reconstruct the FMFA measurement from the 3D volume was 2 minutes. 

## 4. Discussion 

The principal finding from this study was of significantly lower 2D and 3D FMFA measurements in Caucasian fetuses compared with those from individual Asian sub-groups. The 3D findings support those of Jeon et al. [4] but are at odds with Chen et al., where no significant difference between Chinese and Caucasian 3D FMFA measurements was found [5]. This is the first study to describe the 2D FMFA measurement characteristics by ethnicity and to compare ethnic Asian sub-groups. Whilst no difference was found between the individual Asian sub-groups in 2D measurements, the findings on 3D imaging showed significant differences between all sub-groups. These differences may indicate the need to establish ethnic-specific reference ranges for both 2D and 3D imaging.

There was a significant difference between 2D and 3D FMFA measurements in the Caucasian and South Asian sub-groups but not between the north and East Asian groups. These findings in part agree with our previous results identifying 2D FMFA measurements to be larger than those obtained in 3D by 0.89° [3]. The variable findings of the current study may be influenced by the fact that fewer than 90% of north and East Asian fetuses had successful 2D FMFA measurements, whilst 100% and 90% of Caucasian and South Asian fetuses, respectively, had both 2D and 3D FMFA measurements performed. The high exclusion rate of 2D FMFA measurements due to operator inexperience and the fact that 2D FMFA measurements are reported to be more difficult to perform without adequate visualization of fetal landmarks could also contribute to these findings [8].

Sonek et al. [7] found that the mean FMFA in Down syndrome (trisomy 21) fetuses was 88.7° and that 69% of those fetuses had a FMFA measurement above the 95th centile (which equated to 85° across the gestational age range of 11 + 0 to 13 + 6 weeks) [2]. In this study, 31% of 2D and 26% of 3D in the Caucasian group FMFA measurements fell above this 95th percentile threshold value, agreeing with an earlier study [1]. The median 2D and 3D FMFA measurements in the Asian sample were all greater than the trisomy 21 mean of 88.7°, with a significant proportion (47–77% in 2D imaging; 49–78% in 3D imaging) of the fetuses from each Asian sub-group exceeding the threshold value of 85°. In the combined Asian group, 61% (2D) and 57% (3D) expressed a FMFA measurement greater than 85°. This finding concurs with a recent Korean study where 65.2% of fetuses recorded a 3D FMFA measurement of greater than 85°. This further highlights the need for precision in the FMFA measurement, given the small FMFA increase above the gestation-specific median value in Down syndrome fetuses [9].

The study has several salient strengths, including its robust prospective design, careful data management, and statistical analysis. However, the study also has some limitations. One potential limitation of the study is the small sample size, particularly for the south and East Asian groups. Arguably, the most important limitation of the study is the high exclusion rate due firstly to variable operator training and secondly to maternal and fetal factors that preclude adequate imaging and measurement. Although similar levels of exclusion have been found in previous studies [2, 10, 11], this may mask the true variability of the measurement. Even with highly experienced operators, FMFA measurement is not easy to perform. A study of 3D offline FMFA measurement by accredited NT practitioners concluded that competence was achieved after a median of 90 examinations [9]. In a recent study by the current authors, it took a second experienced operator 40 patients to achieve acceptable reproducibility of FMFA measurement in both 2D and 3D [8]. 

Most of the limited literature on this subject relates to the 3D method of FMFA measurement as the gold standard. There are limitations to performing the measurement only in 3D, including the nonavailability of 3D technology across the ultrasound community and the need for computer software and network connections to allow offline analysis. Furthermore, offline analysis necessarily delays the dissemination of relevant information to the patient and the referring practitioner. 

Given the technical difficulties inherent in the examination and the high exclusion rates it is unlikely that FMFA assessment could be easily incorporated into routine first Down syndrome screening in an Asian population. A larger, multicenter study across geographically different regions may be necessary to further assess the utility of both 2D and 3D FMFA measurements in an Asian population.

## Figures and Tables

**Table 1:** FMFA medians, range, and number for both 2D and 3D according to ethnicity with Wilcoxon matched pairs signed-ranks test.

		Caucasian (C)	North Asian (NA)	South Asian (SA)	East Asian (EA)	Comparison	*z*	*P*
2D	Median	86.8°	89.2°	89.0°	88.9°	C-NA	−6.21	<0.001
						C-SA	−3.68	<0.001
	Range	76.2°–97.3°	84.3°–95.4°	84.0°–93.4°	86.2°–95.7°	C-EA	−3.54	<0.001
						NA-SA	0.44	0.66
	*n*	220	95	46	29	NA-EA	−1.05	0.29
						SA-EA	−0.53	0.60

3D	Median	86.0°	89.5°	88.9°	89.8°	C-NA	−8.18	<0.001
						C-SA	−4.20	<0.001
	Range	76.7°–95.7°	85.2°–94.0°	82.8°–92.4°	86.8°–95.3°	C-EA	−4.87	<0.001
						NA-SA	2.10	0.04
	*n*	220	108	51	36	NA-EA	−1.94	0.05
						SA-EA	−2.53	0.01

	*z*	3.94	−1.74	2.01	−0.82			
	*P*	<0.001	0.08	0.04	0.41			
